# FGF19 subfamily members: FGF19 and FGF21

**DOI:** 10.1007/s13105-019-00675-7

**Published:** 2019-03-29

**Authors:** Katarzyna Dolegowska, Malgorzata Marchelek-Mysliwiec, Monika Nowosiad-Magda, Michal Slawinski, Barbara Dolegowska

**Affiliations:** 1 Clinical Department of Nephrology, Transplantology, and Internal Medicine, Pomeranian Medical University, Szczecin, Poland; 2 Department of Immunology Diagnostics, Pomeranian Medical University, Szczecin, Poland; 3 Department of Laboratory Diagnostics, Independent Public Clinical Hospital No. 2, Pomeranian Medical University, Szczecin, Poland; 4 Department of Laboratory Medicine, Pomeranian Medical University, Szczecin, Poland

**Keywords:** FGF19, FGF21, Bile acids, Metabolism regulation, Klotho

## Abstract

Fibroblast growth factors (FGF) constitute a large family of proteins with pleiotropic effects on development, organogenesis, and metabolism. The FGF19 subclass includes growth factors circulating with the blood referred to as endocrine FGF. Representatives of the FGF19 subclass, including FGF19, FGF21, and FGF23, act via FGFR receptors. The proteins of FGF19 subfamily influence the enterohepatic circulation of bile, participate in glucose and lipid metabolism regulation, and maintenance of phosphorus and vitamin D3 homeostasis. FGF19 and FGF21 are activated under different physiological and pathological conditions.

## Introduction

**Fig. 1:**
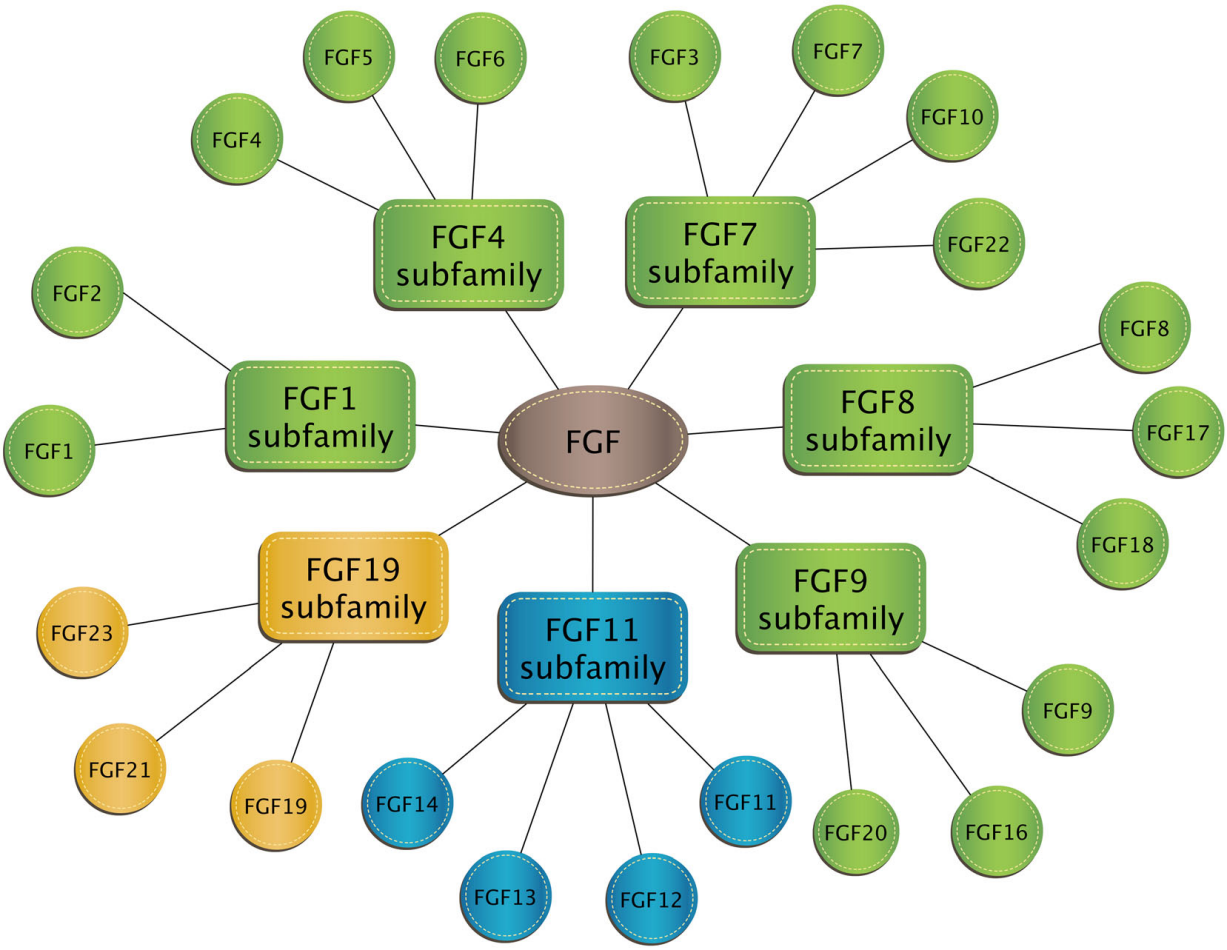
Classification of human fibroblast growth factors into seven subclasses. Green color indicates subclasses containing paracrine and/or autocrine factors; blue = nuclear FGF, yellow = endocrine FGF

Fibroblast growth factors (FGF) constitute a large family of proteins with pleiotropic effects on development, organogenesis, and metabolism. Despite the name, not all FGF stimulate fibroblast activity. Inclusion in the FGF family is based on structural similarity. The FGF family includes 22 agents that have been classified into seven subclasses based on phylogenetic similarity. The FGF1 subfamily contains two typical growth factors: FGF1 (also referred to as acidic fibroblast growth factor) and FGF2 (basic fibroblast growth factor). Factors from the FGF1, FGF4, FGF7, FGF8, and FGF9 subclasses are characterized by paracrine and/or autocrine activities. The FGF11 subclass consists of so-called nuclear FGFs that operate intracellularly and do not bind to the fibroblast growth factor receptor (FGFR). In turn, the FGF19 subclass includes growth factors circulating with the blood referred to as endocrine FGF [20, 49, 75] (Fig. 1).

Representatives of the FGF19 subclass, including FGF19, FGF21, and FGF23, act via FGFR receptors. They show activity on FGFR1c, 2c, 3c, and 4 receptors, while they can not bind to FGFR1b, 2b, and 3b receptors. FGFR expression is diverse, but shows similarity in some tissues. Most FGF ligands act autocrinally and/or paracrinally [1]. Most FGF bind to corresponding cell membrane receptors of tyrosine kinase activity through high-affinity heparin fixed to the endothelium surface. In contrast, the FGF19 subclass does not have a heparin-binding domain. This characteristic feature allows FGF19, FGF21, and FGF23 proteins to evade paracrine and/or autocrine effects. Most FGF ligands act autocrinally and/or paracrinally. These FGFs bind to corresponding cell membrane receptors of tyrosine kinase activity through high-affinity heparin fixed to the endothelium surface. In contrast, the FGF19 subclass does not have a heparin-binding domain [1, 10, 15]. The FGF19 subfamily shows a weak affinity for heparan sulfate (HS), which allows them to diffuse easily from the site of secretion into the blood where they can work as hormones. This “FGF endocrine group” consist of FGF19, FGF21, and FGF23 [10, 15]. The FGF21 may be also autocrine factor that controls beige adipocytes appearance and activity [15]. Moreover, FGF21 is secreted autocrinally by cardiomyocytes via the Sirt1-PPARα pathway. It protects against hypertrophy, metabolic dysregulation, and activation of inflammatory pathways [10].

Factors of the FGF19 subclass show a low affinity for FGFR when compared to other growth factors. They require the presence of a co-receptor—Klotho protein—to elicit activity on cells [16, 43, 72, 75].

Klotho proteins belong to a group of transmembrane proteins which consists of the following subfamilies: α-Klotho, β-Klotho, and γ-Klotho. α-Klotho is a protein needed to induce a biological effect by FGF23, while β-Klotho is necessary for the activity of FGF19 and FGF21 factors. Endocrine growth factors show low affinity for FGFR receptor or Klotho protein itself, but high for the FGFR-Klotho complexes. FGFR expression can be found in most tissues and cells, while tissue-specific Klotho expression determines target tissues for the FGF19 subfamily. α-Klotho can be found in kidneys and parathyroid glands where it forms complexes with FGFR 1c, 3c, and 4, which serve as high affinity receptors for FGF23. β-Klotho forms complexes with FGFR 1c and 4 in liver and adipose tissue, which bind to FGF19 and FGF21. The γ-Klotho and FGFR 1b, 1c, 2c, and 4 complexes that bind FGF19 can be found in the eye, connective tissue, and the kidney [20, 75].

**Fig. 2:**
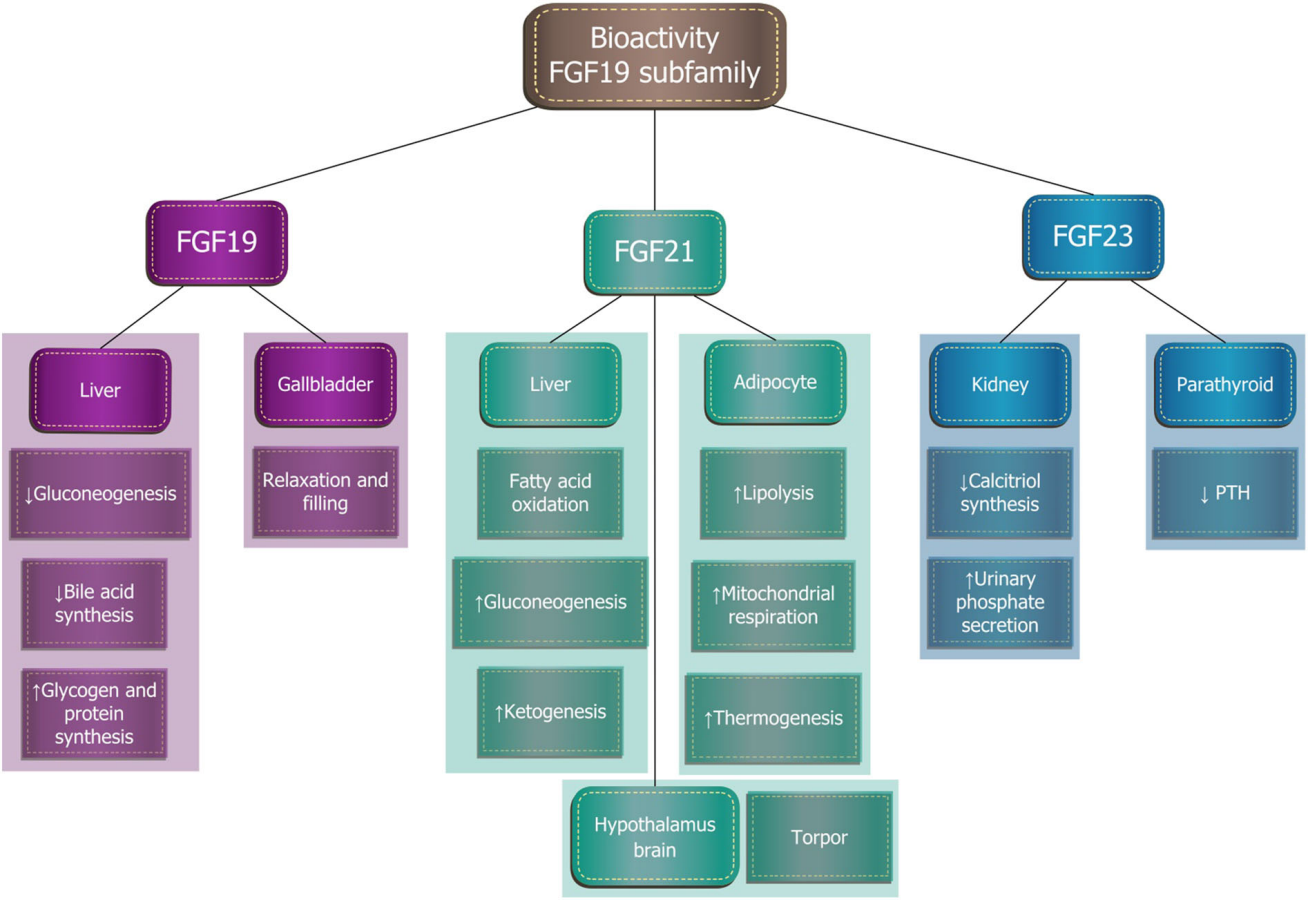
Biological effects of fibroblast growth factors of the FGF19 subfamily

The proteins of FGF19 subfamily influence the enterohepatic circulation of bile, participate in glucose and lipid metabolism regulation, and maintenance of phosphorus and vitamin D3 homeostasis (Fig. 2). Although FGF19 and FGF21 structures show homology of only 35%, their functions overlap to a large extent and they are involved in the maintenance of a constant body weight and the regulation of carbohydrate and lipid homeostasis. Recent studies have shown that increased expression or administration of exogenous FGF19 increases the synthesis of hepatic glycogen and proteins, and is associated with a lean phenotype in mice. This factor increases glucose tolerance and insulin sensitivity of tissues, reduces gluconeogenesis, and increases the amount of brown adipose tissue. FGF19 is synthesized after every meal, especially if the meal is rich in fat. It participates in the negative feedback control of bile acids synthesis and secretion. In turn, FGF21 is synthesized and secreted by the liver during starvation. It impacts yellow adipose tissue, where it promotes lipolysis, fatty acid oxidation in the mitochondria, and “browning” of adipose tissue, the appearance and activation of brown adipose tissue cells. FGF21 correlates with browning in white adipose tissue (iWAT) and the induction of UCP1. Treatment of human pre-adipocytes with FGF21 induces thermogenic gene expression and activity [15]. The current research assesses the possibility of using FGF21 in the treatment of obesity [20, 60, 75].

## Fibroblast growth factor 19

FGF19 was first discovered in 1999 in the human brain in the course of embryonic and fetal development. FGF15, present in animals, is an ortholog of FGF19 and occurs in the central nervous system of rodents, where it stimulates the differentiation of mature neurons. Many animal studies have confirmed the therapeutic potential of FGF19 in the treatment of metabolic disorders, such as obesity or diabetes. However, currently, the most research focuses on possible mitogenic effects of FGF19 [75].

### Bile acids homeostasis

Bile acids are compounds with a strong emulsifying effect, which are synthesized in the liver from cholesterol. After each meal, the gallbladder contracts and releases bile acids into the intestine to enable the solubilization and absorption of lipids and fat-soluble vitamins. Because of their toxicity, their synthesis must be strictly regulated [25, 28, 68]. Cholesterol 7α-hydroxylase (CYP7A1) is an enzyme that catalyzes the regulatory reaction of bile acid synthesis and is the main target of a negative feedback control. Bile acids inhibit the transcription of the CYP7A1 gene and thereby reduce their own synthesis. The gene is induced by cholesterol and oxysterols (oxysterols influence has so far been proven only in rodents). Bile acids regulate the expression of the CYP7A1 gene via the nuclear receptor farnesoid X receptor (FXR). In FXR-deficient mice, inhibiting feedback is absent and CYP7A1 expression is increased [56].

**Fig. 3:**
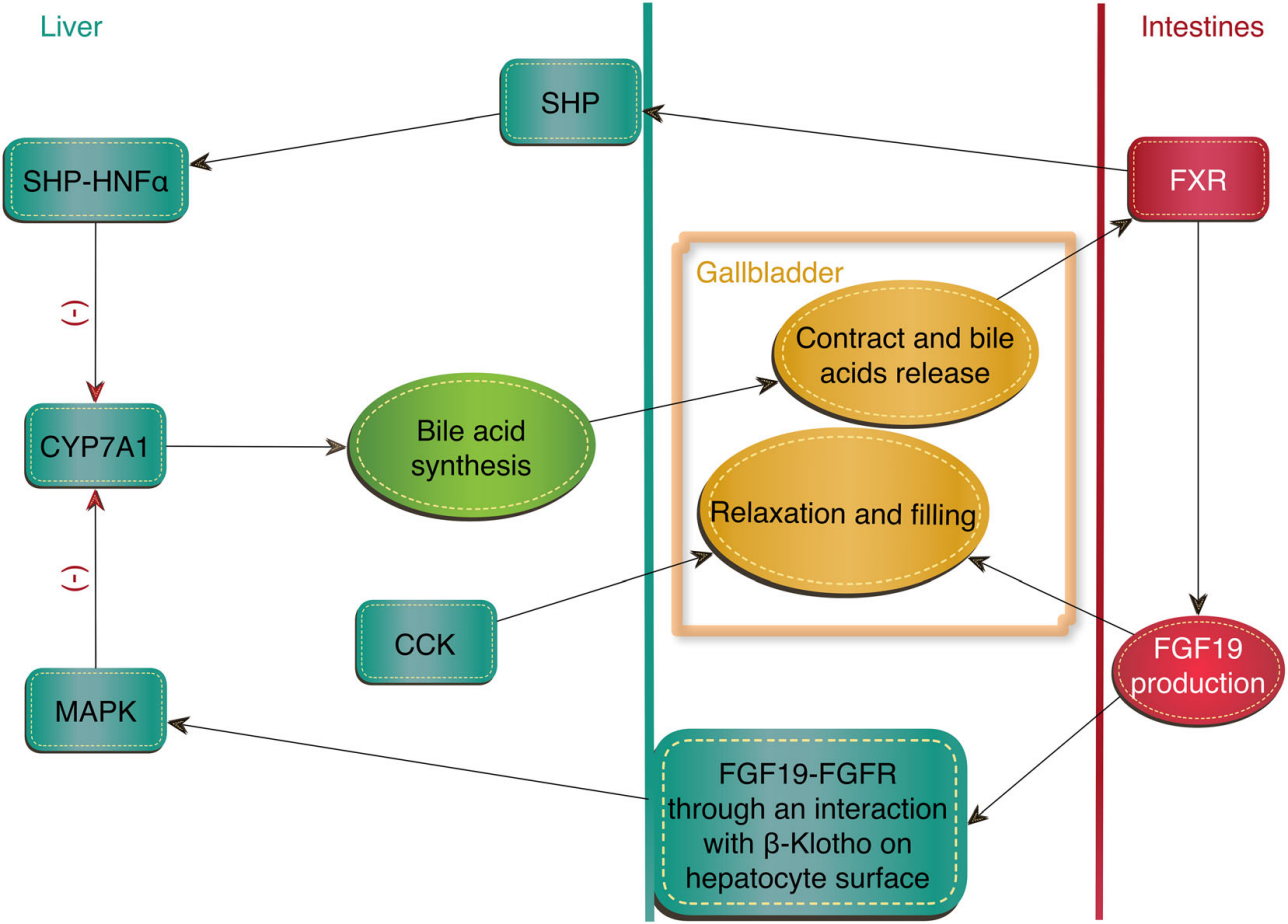
Regulation of bile homeostasis through FXR nuclear receptor. CCK = cholecystokinin, CYP7A1 = cholesterol 7α-hydroxylase, FXR = farnesoid X receptor, MAPK = mitogen-activated protein kinases, SHP = small heterodimer partner, SHP-HNFα = hepatocyte nuclear factor and small heterodimer partner complex

FXR receptor induces an expression of small heterodimer partner (SHP) in the liver. Unlike most nuclear receptors, SHP does not have a DNA-binding domain but can interact with many proteins. Hepatocyte nuclear factor (HNF-α) that binds and activates CYP7A1 is one of them. The formation of HNF-α and SHP complex prevents the interaction of HNF-α with co-activators, and their dissociation causes repression of the CYP7A1 gene. However, this is not the only regulatory mechanism of FXR. Negative feedback requires a synthesis of FGF19 in the small intestine, triggered by FXR activation. In humans, the highest concentration of FGF19 in the blood occurs 90–120 min after a meal. FGF19 activates the FGFR4 receptor on the hepatocyte surface through an interaction with β-Klotho and stimulates the MAP kinase signaling pathway, inhibiting CYP7A1 expression in the liver (Fig. 3). Patients with impaired fatty acids absorption showed reduced intestinal production of FGF19 [2, 9, 18, 19, 28, 45, 56, 75].

FGF19 also affects the gallbladder—causing its relaxation, thus the opposite effect of cholecystokinin (CCK), which causes gallbladder contraction and bile release [28]. Research conducted on FGF15^−/−^, FGFR4 ^−/−^, and β-Klotho^−/−^ mice found that their gallbladders are much smaller. After an administration of FGF15 to the FGF15^−/−^ mice, gallbladder sizes returned to normal [9].

### Metabolism of carbohydrates

Metabolism of carbohydrates is regulated by many factors, including insulin and glucagon, which are early reacting hormones. Insulin is excreted after a meal and it stimulates hepatic synthesis of glycogen, lipogenesis, and inhibits gluconeogenesis. FGF19 is a late-reacting hormone which also stimulates glycogen synthesis, inhibits gluconeogenesis, but does not increase lipogenesis [28, 75].

FGF19 reduces gluconeogenesis by inhibiting the expression of the PGCα transcription factor and its target genes: glucose-6-phosphatase (G6Pase) and phosphoenolpyruvate carboxykinase (PEPCK). FGF15^−/−^ and FGFR4^−/−^ mice showed increased mRNA of PGC1α, G6Pase, and PEPCK. In addition, these mice showed higher glucose concentrations after stimulation of gluconeogenesis with pyruvate or lactate than wild-type mice [28, 75]. Insulin reduces expression of genes involved in gluconeogenesis by promoting Akt-dependent phosphorylation and further degradation of FOXO1. FOXO1 is a transcription factor involved in the induction of gluconeogenesis gene expression during fasting. FGF19 does not change the phosphorylation of FOXO1 because it does not activate the PI3K/Akt pathway. Instead, FGF19 regulates the transcription of a factor which is involved in the expression of gluconeogenesis genes—CREB (cAMP response element-binding protein).

FGF19 reduces phosphorylation of CREB and inhibits CREB binding to PGC1α promoter. By reducing PGC1α transcription, the amount of PGC1α transcription factor associated with G6Pase and PEPCK promoters is decreased [28]. FGF19 induces phosphorylation of glycogen synthase kinase 3α (GSK3α) and kinase GSK3β, which results in, among others, phosphorylation and inactivation of glycogen synthase (GS). Phosphorylation suppresses GSK3α/β activity, thus FGF19 reduces phosphorylation of GS, which induces its activity and leads to an increase in the amount of glycogen in the liver of mice. FGF15^−/−^ animals have less glycogen in the liver and less effectively reduce glucose concentration after its administration compared to the wild type. Administration of FGF19 significantly increases the glucose tolerance in FGF15^−/−^. FGF19 also stimulates glycogen synthesis in mice with streptozotocin-induced diabetes with very low insulin levels. That study showed that FGF19 induces glycogen synthesis independently of insulin [28].

### Protein synthesis

FGF19 enhances protein synthesis by increasing phosphorylation of eukaryotic initiation factors 4B, (eIF4B) and eIF4E, which are parts of the eIF4F complex. eIF4F mediates the binding of mRNA to a ribosome. FGF19 also increases phosphorylation of the ribosomal protein S6 (rpS6) subunit, which leads to further stimulation of protein synthesis [28].

### Lipid metabolism

A study performed in Japan showed that in FXR receptor knockout mice, hepatic lipid levels are higher than in wild-type mice. Administration of recombinant FGF19 prevented fat deposition and decreased alanine aminotransferase (ALT) activity and concentrations of hepatic lipids, including triglycerides and free fatty acids. The treatment significantly reduced the transcription of genes of proteins associated with lipogenesis, including acetyl-CoA carboxylase (ACC), Cd36, Srebp-1c, SCD1, and Cyp7a1 [42]. Tomlinson et al. [59] found that the body weight of FGF19 transgenic mice decreased significantly despite receiving more food than control mice. In addition, transgenic mice appeared to be resistant to a high-fat diet. Research on the mechanism of action has shown that FGF19 reduces the ability of insulin to stimulate fatty acid synthesis. FGF19 inhibits the expression of the transcription factor - sterol regulatory element binding protein-1c (SREBP-1c), which is a factor associated with the expression of insulin-regulated genes necessary for glucose metabolism and lipids and fatty acid synthesis. Moreover, FGF19 activity increases the expression of SHP, which inhibits the expression of lipogenic enzymes through the mechanism associated with SREBP-1c [6].

Interestingly, Wu et al. [66] showed that treatment with FGF19 increases triglycerides and cholesterol blood levels in mice with obesity resulting from diet. It has been proposed that, depending on conditions, FGF19 may both increase and decrease lipid concentration. This dual action can be attributed to the binding of FGF19 to different receptors in target tissues. For example, FGF19 induces lipolysis by activating FGFR1c receptor present mainly in adipose tissue and other tissues except the liver. On the other hand, FGF19 induces lipogenesis through FGFR4 receptor and inhibits hepatic synthesis of fatty acids [75].

### Obesity

FGF19 and FGF21 play an important role in the regulation of glucose and lipid metabolism. Clinical studies show that FGF19 concentration in the blood of obese patients is lower than in healthy subjects [21, 75]. FGF19 concentration is also reduced in obese adolescents with non-alcoholic fatty liver disease (NAFLD), when compared to healthy subjects. Interestingly, there is a correlation between low FGF19 levels during fasting and the likelihood of NAFLD occurrence in obese children [65]. However, Schreuder et al. showed that in patients with NAFLD and insulin resistance, FGF19 synthesis is preserved, while liver response to FGF19 is impaired [54].

The negative correlation between FGF19 and obesity has also been confirmed in animal studies. Administration of human recombinant FGF19 to mice in which obesity was induced by high-fat diet resulted in a dose-dependent significant decrease in body weight and blood glucose concentration. FGF19 has been shown to increase leptin receptor expression and to reduce the expression of acetyl-CoA 2 carboxylase in the liver, which leads to an increase in lipid oxidation and a decrease of hepatic triglyceride levels. In addition, after a prolonged administration of FGF19, the expression of genes associated with the activation of brown adipose tissue increases [12, 75].

### Diabetes

Transgenic mice bearing the FGF19 gene show an increased sensitivity to insulin compared to the wild type [59]. Similarly, the administration of exogenous FGF19 prevents the development of glucose metabolism disorders. In mice with obesity genetically induced by ablation of brown adipose tissue or lack of leptin, FGF19 prevented or reversed diabetes [12]. Recent studies have demonstrated that FGF19 administered to cerebral ventricles results in insulin-independent decreasing glucose levels in obese mice and increasing insulin sensitivity in mice on a high-fat diet. This indicates regulation of blood glucose level by FGF19 via the central nervous system [75].

In diabetic people, the change in FGF19 concentration is ambiguous [75]. Brufau et al. found similar concentrations of FGF19 in healthy and diabetic patients [7]. In contrast, pregnant diabetic patients showed significantly lower FGF19 concentrations when compared to healthy pregnant women [61, 62]. This is consistent with the results obtained in people with diabetes and metabolic syndrome in whom FGF19 concentration negatively correlated with BMI, triglycerides, HDL cholesterol, CRP, and glycated hemoglobin levels [5]. The number of publications on the resolution of type 2 diabetes after the gastric reduction surgery is increasing; however, the mechanism is unknown [24]. Before the surgery, lower concentrations of FGF19 and fatty acids were observed in people with diabetes compared to nondiabetic patients regardless of BMI. After the surgery, a gradual increase in FGF19 and fatty acids concentrations was observed in all patients; however, it was greater in people with diabetes [5].

### Kidney diseases

Reiche et al. [51] have shown that FGF19 serum concentration was 1.5 times higher in hemodialyzed patients when compared to healthy subjects. In addition, FGF19 was positively correlated with adiponectin and negatively with CRP concentrations. In the end stage of chronic kidney disease, an impaired post-meal FGF19 response was observed, which was partially normalized by therapy with antioxidants [34].

## Fibroblast growth factor 21

The *fgf21* gene was first discovered in mice and later identified in the human genome during the search for the gene homolog [10]. The human FGF21 is composed of 209 amino acids and the mouse analog has 210. Both mouse and human factors show 75% homology [30]. FGF21 is expressed predominantly in the liver and adipose tissue and in smaller amounts in the skeleton, muscle, heart, kidneys, and testes. Kharitonenkow et al. [26] have shown that FGF21 increases glucose uptake by mouse 3T3-L1 cells, which, under proper conditions, exhibit a phenotype similar to adipocytes. In addition, FGF21 increases glucose tolerance and tissue insulin sensitivity and reduces blood glucose level. Similar effects have been observed in non-human primates. An overdose of FGF21 does not lead to hypoglycemia as with insulin. FGF21 also does not show carcinogenic potential [10, 20, 26, 75].

There are many sites of FGF21 synthesis: white adipose tissue, brown adipose tissue, pancreas, skeletal muscle, and cardiac endothelial cells, but the liver is the most important one. In starving mice, a marked increase in FGF21 hepatic expression and plasma concentration was observed, which was then suppressed after a meal. Peroxisome proliferator-activated receptors α (PPARα) is a transcription factor that controls hepatic FGF21 expression in hunger, as well as lipid metabolism and energy homeostasis. Fenofibrate, a PPARα agonist, is a potent inducer of FGF21 expression in hunger both in the mouse liver and in human primary hepatocytes. Fenofibrate does not work in PPARα-knockout (KO) mice. In case of FGF21 deficiency in mice, the biological activity of PPARα is reduced. It is possible that PPARα, mainly with help of FGF21, mediates in adaptation of metabolism during hunger or after a meal, including ketogenesis, fatty acid oxidation, or gluconeogenesis [10, 17, 37, 47, 75].

**Fig. 4:**
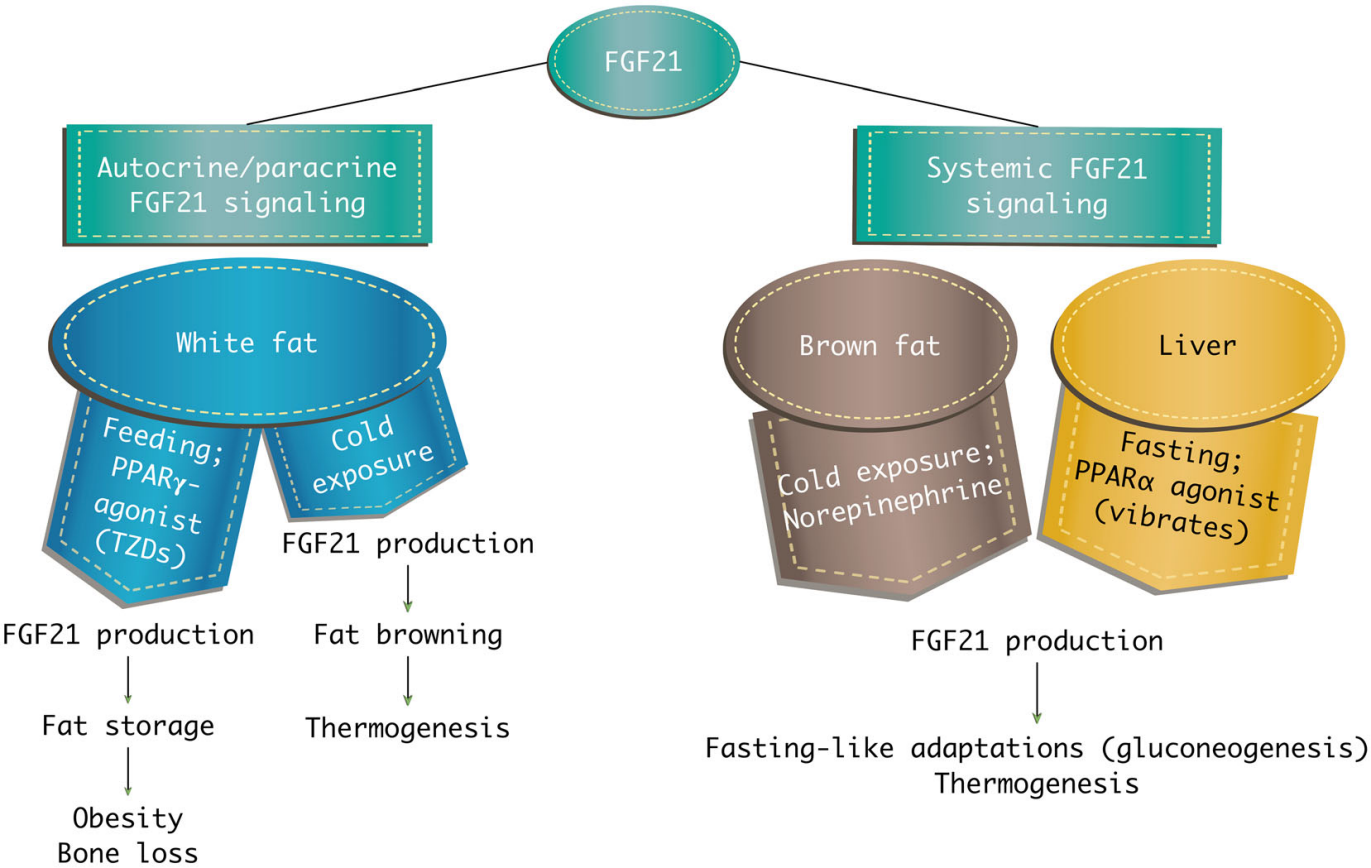
Systemic and local actions of FGF21. PPARα = peroxisome proliferator-activated receptors α, PPARγ = peroxisome proliferator-activated receptors γ, TZDs = thiazolidinediones

In white adipose tissue, FGF21 stimulates glucose entry, regulates lipolysis, increases mitochondrial oxidative capacity, and enhances the effect of peroxisome proliferator-activated receptors γ (PPARγ). It has been also shown that FGF21 influences regulation of thermogenic mechanisms in brown adipose tissue. FGF21 increases glucose transport with GLUT-1, but not with GLUT-4 as insulin does [10]. Mice with the *fgf21* gene deletion (FGF21-KO, FGF21-knockout) do not show PPARγ effects, such as reduction of fat and lipemia, improvement of tissue insulin sensitivity, and increase of lipogenesis. Administration of PPARα activator induces FGF21 expression in the liver, which results in an increase of circulating FGF21. In contrast, activation of PPARγ enhances the FGF21 expression in adipose tissue, but does not lead to an increase of FGF21 circulating in blood. FGF21 secreted from fatty tissue may be inhibited by the extracellular matrix of white adipose tissue [10, 27, 47, 75] (Fig. 4).

FGF21 is considered the missing link between peripheral metabolic tissues and the brain. The brain participates in the control of the body fat content and glucose and lipids homeostasis. FGF21 penetrates the blood-brain barrier in one direction and is present in the cerebrospinal fluid in humans and in the hypothalamus of fasting mice, where it induces ERK1/2 phosphorylation [15, 44]. Recent studies demonstrated that expression of β-Klotho in the nervous system of mice is necessary for many FGF21 activities, involving circadian control of metabolism and female reproductive hormones [15]. In mice with diet-induced obesity and missing β-Klotho co-receptor in the central nervous system, the beneficial effects of FGF21 on the body weight or insulin resistance were not observed [15, 44]. The administration of FGF21 directly into the cerebrospinal fluid increases energy expenditure and reduces insulin resistance in obese rats [10, 70].

PPARα regulates the FGF21 expression so it is probably engaged in signal transmission from the liver to the brain. In response to a reduction of food amount, physiological activity is reduced causing lethargy, which in animals manifests as a decrease in body temperature and metabolic rate. Administration of bezafibrate, a PPARα agonist, to mice induces a temporary lethargy-like state during which FGF21 production in the liver increases. Both transgenic expression and administration of FGF21 induce mice into a lethargic state. Similarly, intraventricular administration of neuropeptide Y (NPY) induces lethargy-like hypothermia, resembling natural lethargy in hamsters. Since bezafibrate also stimulates NPY production, it is possible that PPARα is involved in the control of lethargy and circadian rhythm via the FGF21-NPY axis [10].

Many animal studies have demonstrated FGF21 protective effects against various pancreatic damages and β cell dysfunctions. FGF21 KO mice are more susceptible to cerulein-induced pancreatitis (CIP), while FGF21 transgenic mice are resistant to acute pancreatitis. The FGF21 protective effect results from its ability to activate extracellular signal regulated kinases 1 and 2 (ERK1/2) in pancreatic stellate cells. Expression of FGF21 occurs in pancreatic islets in humans, rats, and mice, as well as rat pancreatic primary β and INS-1E cells. Short-term administration of FGF21 reduces insulin levels in both healthy and db/db mice (diabetes mice). In contrast, long-term FGF21 administration increases the insulin secretion in db/db mice. FGF21 inhibits glucagon secretion from isolated rat islets and reduces glucagon concentration in mice. FGF21 does not influence pancreatic islet cells proliferation, but it activates ERK1/2 and Akt signaling pathways in rat pancreatic islets, protecting them against glucotoxicity and cytokine-induced apoptosis [10, 57].

Increased FGF21 expression occurs as a result of various types of stress. It may be a chemical stress induced by acetaminophen, dioxin, cerulein, or phenylephrine. A similar effect is caused by mitochondrial and oxidative stress, tunicamycin, thapsigargin, and phenylephrine, which induce stress in the endoplasmic reticulum, as well as by environmental factors such as cold, starvation, and overfeeding. This suggests that FGF21 is a key regulator in body adaptation process to various types of stress [75].

### Mechanism of FGF21 operation

Many studies indicate that a β-Klotho co-factor determines the metabolic activity of FGF21. FGF21 initiates its action by binding to FGFR receptor in the presence of β-Klotho co-factor, which has been discovered in the liver and adipose tissue. FGF21 induces ERK1/2 phosphorylation in the liver and adipose tissue, but does not show this effect in the heart or skeletal muscles, where β-Klotho is not present. This signal pathway suggests that the metabolic effect of FGF21, i.e., glucose concentration reduction occurs through the liver and adipose tissue [3, 10, 15, 17, 20].

There are many mechanisms that could be related to the transcriptional or post-translational regulation of FGF21 activity [17, 26]. FGF21 elicits an effect by mediating post-translational modifications. In contrast to the peroxisome proliferator-activated receptor (PPARγ) agonist and metformin, FGF21 leads to a rapid insulin sensitization within 1 h. Such fast action may indicate post-translational mechanisms without the need for new protein synthesis. However, subsequent effects of FGF21 action may be associated with mechanisms of gene transcription and protein synthesis. The ERK pathway is activated by FGF21 and regulates gene expression by controlling the action of transcription factors and changing transcription of genes [15, 17, 70].

It has been shown that in 3T3-L1 adipocytes, FGF21 increases the expression of GLUT1, which results in increased glucose uptake. This effect disappears under the influence of protein synthesis inhibitors, which suggests that FGF21 also acts through transcriptional mechanisms. In addition, FGF21 shows the ability to regulate the expression of the lipogenic gene by reducing the amount of sterol regulatory element binding protein-1 (SREBF1) transcription factor in the nucleus [10, 26, 31, 50, 64, 69, 70].

### Lipid metabolism

Incubation of hepatocytes with fatty acids or intravenous lipids administration stimulate the secretion of FGF21 [38]. Studies have shown that FGF21 levels are positively correlated with obesity and hepatic steatosis degree. They demonstrate that increased FGF21 concentration may be considered as an adaptive mechanism protecting the body against lipotoxicity. Inagaki et al. [22] showed that the amount of liver triglycerides and their blood concentration is significantly reduced in FGF21 transgenic mice compared to wild-type mice. Reduction of FGF21 level in mice on a ketogenic diet leads to hepatic steatosis, lipemia, and a decrease of serum ketone concentration [4, 75]. Transgenic mice also have notably smaller adipocytes [22].

The FGF21 factor induces lipolysis in adipose tissue, especially via hormone-sensitive lipase and TG lipase. Furthermore, FGF21 also induces fatty acids β-oxidation by increasing the expression of 3-hydroxyacyl-CoA dehydrogenase, 1α carnitine palmitoyltransferase, acyl-CoA oxidase, CD36, and the AMPK-SIRT1-PGC1α signaling pathway mediated by adiponectin. Another study has shown that FGF21 induces adipocyte differentiation and lipogenesis in obese mice. FGF21 may show opposite regulatory effects on lipid metabolism and stimulate or inhibit lipolysis in adipocytes [75].

### Heat production in adipose tissue

White adipose tissue (WAT) is the main tissue involved in fat storage. Brown adipose tissue (BAT) participates in thermogenesis. Fatty acids released from white adipose tissue during lipolysis are a source of heat in mitochondria thanks to the UCP1 uncoupling protein. UCP1 generates a thermogenic effect through elimination of the electric potential difference across the mitochondrial membrane and transformation of this energy into heat instead of ATP. However, recent studies have shown that over prolonged cold exposure, white adipose tissue can transform into brown-like, or beige adipose tissue [11, 75]. FGF21 acts autocrinally/paracrinically on adipocytes, which leads to further induction of FGF21 expression [54]. Fisher et al. [11] demonstrated that FGF21-KO mice show impaired ability to convert WAT into beige adipose tissue and to adapt to low temperatures. Administration of exogenous FGF21 increases the body ability to defend against chronic cold by increasing the expression of UCP1 and other thermogenic genes in adipose tissue. FGF21 regulates this process, at least partially, by increasing the peroxisome proliferator-activated receptor-γ co-activator 1α (PGC1α) protein level in adipose tissue, regardless of mRNA expression [11, 29, 47, 54, 75]. Studies on the relationship between WAT and an FGF21 pharmacological action in metabolism regulation concluded that the therapeutic effect of FGF21 on carbohydrate and lipid metabolism disorders is independent of the transformation of white into brown adipose tissue. They confirmed that after administration of mimetic FGF21, BAT activity and UCP1 expression were significantly increased both at 21 °C and 30 °C, whereas transformation to beige tissue occurred only at 21 °C. However, exogenous FGF21 or mimetic FGF21 induce similar effects, such as increased energy expenditure without altering the food intake, weight loss, and regulation of glucose and lipid levels. This is achieved in both temperatures, both in wild-type and in UCP-1-KO mice [52, 60, 75].

Many effects of FGF21 on WAT and BAT require action on the central nervous system. FGF21 stimulates release of corticoliberin from the nervous system and activates the sympathetic system, which leads to browning of the white adipose tissue, oxidation of fatty acids in the brown adipose tissue, thermogenesis, and lipolysis necessary for ketogenesis [15, 36, 44]. The research reveal that some effects of FGF21 disappear in mice lacking β-Klotho in the nervous system, while others, like the role of FGF21 in lowering of blood glucose are preserved and disappear only in the absence of this co-receptor in the whole body [36, 44].

### Nutritional states

Unlike FGF19, FGF21 maintains glucose homeostasis in different nutritional states. During fasting/starvation FGF21 induces lipolysis, fatty acid oxidation, ketogenesis, and gluconeogenesis and increases insulin sensitivity [3, 10, 15]. Fibroblast growth factor 21 administration reduces fasting plasma glucose, triglyceride, low-density lipoprotein cholesterol (LDL), and insulin levels in mice. It also increases high-density lipoprotein cholesterol (HDL) concentration and causes weight loss [10, 15, 26]. Inagaki and al. [22] showed that the ketogenic effect in starvation in mice with increased expression of the *fgf21* transgenic gene (FGF21-TG) and in wild-type mice is comparable [22, 23, 75]. In human plasma, FGF21 level is increased after prolonged fasting (> 7 days) [14].

Previous in vitro studies showed that FGF21 induces glucose uptake in various cell lines, probably by increasing GLUT-1 expression and activity [10, 15, 63]. Treatment with insulin and FGF21 induces a synergistic effect that reduces glucose levels by GLUT-4 and GLUT-1 activation in adipocytes [26]. FGF21 has no effect on plasma membrane translocation of the transporter GLUT4, but induces the expression of GLUT1 through transcriptional activation to induce glucose transportation [10]. FGF21 prevents insulin intolerance caused in skeletal muscles and kidneys. Interestingly, the tissues mentioned lack the FGFR1 receptor or β-Klotho protein, which suggests indirect FGF21 influence. Recent studies showed that FGF21 stimulates expression of adiponectin in adipose tissue and suppresses SREBP-2 in the liver [10, 61, 75].

### Diabetes

The FGF21 levels increased in type 2 diabetes and is positively correlated with hypertension, hyperglycemia, glycated hemoglobin level, insulin resistance, and hsCRP level [10]. Multiple studies have been done to determine the relationship between FGF21 and type 2 diabetes mellitus [10, 53, 75]. A large Chinese prospective study has found that increase of FGF21 levels positively correlated with hyperglycemia and dyslipidemia in pre-diabetic subjects of normal phenotype [8]. However, FGF21 level in the case of type 1 diabetes and latent autoimmune diabetes of adults (LADA) was significantly lower than in a healthy control group [61, 67]. The diminishing effect on FGF21 level in type 1 and LADA diabetes is probably caused by insulin deficiency, which acts as an inducer of hepatic FGF21 [53, 75].

A functional study has shown that an administration of exogenous FGF21 to diabetic mice resulted in decreased blood glucose and lipids, improved insulin sensitivity, weight loss, increased fat consumption, and energy expenditure [75]. As FGF21 has been shown to reduce glucose and lipid levels, it has been suggested as a potential therapeutic agent for the treatment of diabetes, obesity, and dyslipidemia [3]. Administration of the FGF21 analogue to people suffering from obesity and type 2 diabetes caused beneficial effects on some disorders associated with diabetes; however, further studies with the analogue are needed [3, 13]. Therefore, FGF21 is currently considered as a therapeutic option but also as a predictive marker for the development of type 2 diabetes.

Since reduced FGF19 level is a contributing factor in the development of gestational diabetes, it created much interest in understanding the role of FGF21 [61, 62]. Stein et al. [58] observed similar blood level of FGF21 in women who are healthy and those suffering from gestational diabetes; however, it was positively correlated with markers of insulin resistance and dyslipidemia, including triglycerides, leptin, adiponectin, and HDL. Although studies suggest that reduced FGF19 level may play a role in the pathophysiology of gestational diabetes, the increased FGF21 level may be a response to the disease [58, 61, 62].

### Obesity

Obese, diabetic mice, as well as people suffering from obesity, show elevated FGF21 levels [3]. Patients with NAFLD also show a high FGF21 level, which is positively correlated with intrahepatic triglycerides [33, 39]. Mouse studies have shown that high-fat diet-induced obesity leads to FGF21 blood level increase. This may suggest obesity-induced resistance to FGF21 due to the negative regulation by β-Klotho. However, recent studies demonstrated that maintenance β-Klotho protein expression in adipose tissue does not increase the adipose tissue FGF21 sensitivity [41]. Whereas sensitivity to FGF21 can be restored by weight loss or by a therapeutic glucose level reduction [33, 75]. Interestingly, the studies in obese diabetic volunteers demonstrate the FGF21 analog LY2405319 causes body weight loss [15, 40].

It has been observed that FGF21 level increases during hunger and in overfeeding in adults as well as in children. Obese children show an elevated blood concentration of free fatty acids that stimulate FGF21 secretion. However, increased FGF21 have a positive correlation with body mass index, waist circumference, body fat mass, insulin, and triglycerides levels and HOMA index. FGF21 may be considered an independent marker of metabolic syndrome and obesity [10, 48].

### Vascular diseases

FGF21 is also produced and secreted by cardiomyocytes and acts as a cardiomyokine [10]. Patients with atherosclerotic carotid arteries disease have been shown to have elevated FGF21 levels that are positively correlated to risk factors such as adverse lipid profile and CRP. High FGF21 levels predicted the incidence coronary artery disease [32]. An in vitro study demonstrated that FGF21 was elevated in cardiac endothelial cells induced by oxidized LDL (oxy-LDL). They were exposed to bezafibrate, a PPARα agonist, which further increased the FGF21 level and inhibited cell apoptosis. In addition, previous studies have observed that FGF21 prevents apoptosis induced by a salt or palmitic acid ester (lipotoxic agent) in H9C2 cells and primary cardiomyocytes, by acting through the ERK1/2–P38 MAPK–AMPK cascade. Similarly, it also protects the heart muscle cells in diabetic patients: it prevents apoptosis in the early stages of a cardiac dysfunction and fibrosis in the end stage [74].

Moreover, FGF21 deletion increases myocardial fatty tissue, likely via positive regulation of NF-E2, an Nrf2 transcription factor, and CD36, which causes increased myocardial oxidative stress and changes in the heart muscle structure and possible development of diabetic cardiomyopathy. FGF21 also protects H9C2 cells (embryonic rat cardiomyocytes) from oxidative stress and apoptosis caused by ischemia-reperfusion injury [71, 75]. Patel et al. [46] demonstrated in the Langendorff system that FGF21 has a protective effect on the myocardium and restores its function also through autocrine/paracrine pathways. However, the protective effect was reduced in obese mice [46]. In addition, FGF21-KO mice were more susceptible than wild-type mice to isoproterenol induced myocardial hypertrophy, which manifested through increased heart mass, ventricular hypertrophy, and cardiac dysfunction. These pathological changes have been reversed with the administration of a FGF21 recombinant [55, 75].

Oxidative stress and energy metabolism in most tissues, including the heart, is controlled by the PGC1α factor. Hypertrophy and inflammatory stimulators inhibit the PGC1α expression. PGCα induction is associated with the activity of FGF21, which limits hypertrophy and inflammation [10].

FGF21 also acts as an antioxidant and prevents accumulation of reactive oxygen species. Secretion of FGF21 into the extracellular space results in amplification of signals for the Sirt1 activation, forming the autocrine loop. In pregnancy, an increase in FGF21 production in the heart as well as in blood level was observed [10].

### Kidney diseases

Animal studies have shown that FGF21 prevents diabetic nephropathy by improving metabolic systems and anti-fibrotic effect. It has been observed that FGF21 has a beneficial effect on the kidneys of mice with type 1 diabetes, because it prevents oxidative stress, inflammation, apoptosis, and fibrosis [73, 75]. Human studies have found FGF21 level correlates with creatinine levels and GFR. Hemodialyzed patients show 15 times higher FGF21 blood concentrations [3, 10]. In patients with chronic and acute kidney disease, FGF21 levels increase as the disease progresses [35, 75].

## Conclusions

Both FGF19 and FGF21 show therapeutic potential due to their regulatory activities. The main subject of the current research is the activity of both FGFs in the presence of metabolic disorders, including obesity or diabetes. In addition, FGF21 has been shown to have protective and regenerative properties on the heart, the kidneys, and the pancreas. FGF19 and FGF21 are also considered as predictors of development of some diseases, for example, type 2 diabetes, non-alkoholic fatty liver disease, atherosclerosis, coronary artery disease, and chronic kidney disease. Further studies are needed to explore therapeutic or predictive properties of both fibroblast growth factors.
